# Structural Analysis of Influenza A Virus Matrix Protein M1 and Its Self-Assemblies at Low pH

**DOI:** 10.1371/journal.pone.0082431

**Published:** 2013-12-16

**Authors:** Eleonora V. Shtykova, Lyudmila A. Baratova, Natalia V. Fedorova, Victor A. Radyukhin, Alexander L. Ksenofontov, Vladimir V. Volkov, Alexander V. Shishkov, Alexey A. Dolgov, Liudmila A. Shilova, Oleg V. Batishchev, Cy M. Jeffries, Dmitri I. Svergun

**Affiliations:** 1 Shubnikov Institute of Crystallography, Russian Academy of Sciences, Moscow, Russia; 2 Belozersky Institute of Physico-Chemical Biology, Moscow State University, Moscow, Russia; 3 Semenov Institute of Chemical Physics, Russian Academy of Sciences, Moscow, Russia; 4 Frumkin Institute of Physical Chemistry and Electrochemistry, Russian Academy of Sciences, Moscow, Russia; 5 Moscow Institute of Physics and Technology, Dolgoprudniy, Russia; 6 EMBL, Hamburg Outstation, c/o DESY, Hamburg, Germany; University of Oulu, Finland

## Abstract

Influenza A virus matrix protein M1 is one of the most important and abundant proteins in the virus particles broadly involved in essential processes of the viral life cycle. The absence of high-resolution data on the full-length M1 makes the structural investigation of the intact protein particularly important. We employed synchrotron small-angle X-ray scattering (SAXS), analytical ultracentrifugation and atomic force microscopy (AFM) to study the structure of M1 at acidic pH. The low-resolution structural models built from the SAXS data reveal a structurally anisotropic M1 molecule consisting of a compact NM-fragment and an extended and partially flexible C-terminal domain. The M1 monomers co-exist in solution with a small fraction of large clusters that have a layered architecture similar to that observed in the authentic influenza virions. AFM analysis on a lipid-like negatively charged surface reveals that M1 forms ordered stripes correlating well with the clusters observed by SAXS. The free NM-domain is monomeric in acidic solution with the overall structure similar to that observed in previously determined crystal structures. The NM-domain does not spontaneously self assemble supporting the key role of the C-terminus of M1 in the formation of supramolecular structures. Our results suggest that the flexibility of the C-terminus is an essential feature, which may be responsible for the multi-functionality of the entire protein. In particular, this flexibility could allow M1 to structurally organise the viral membrane to maintain the integrity and the shape of the intact influenza virus.

## Introduction

Influenza A viruses are important pathogens that still rank among the major global health problems. The virions of the enveloped influenza viruses contain eight segments of single-stranded negative-sense RNA encoding 11 proteins. One of the most important and abundant proteins is the influenza A virus matrix protein M1, that plays essential structural and functional roles in the virus life cycle [1]–[3]. The M1 protein is membrane associates and forms a rigid matrix layer under the viral envelope utilizing multiple protein-lipid and protein-protein interactions (lipid-M1, M1- haemagglutinin, M1- neuraminidase, M1- ribonucleoprotein particles, M1-M1) [4]–[6].

In intact influenza virions, the M1 protein layer is seen on electron microphotographs as a regularly arranged lattice of rods with the size about 4.0×4.0×6.0 nm, which are densely packed perpendicular to the viral membrane [7]. Harris et al [8], and recently Calder et al [9] showed that in the authentic influenza virions M1 protein forms a well ordered helical layer adjacent to the viral envelope, and the close interaction of M1 with the surrounding envelope determines the virion morphology. Accordingly, the rigid matrix layer of M1 maintains the integrity and shape of the intact influenza virus and structurally organises the virus membrane [2]–[6], [8], [9]. Moreover, acting as an endoskeleton, M1 provides the basement and an anchor point for the HA fusion machinery [5], [6]. These studies revealed an important inherent tendency of the protein to self-associate. In a detailed investigation into the self-assembly of recombinant M1 from *E. coli* and of its sub-fragments, the pH-dependent oligomerization of M1 *in vitro* was shown to be determined by both the N- and C-terminal domains of the protein [10].

The M1 protein consists of 252 amino acid residues with a calculated molecular mass (MM) based on the amino acid sequence of ∼28 kDa. Numerous attempts to crystallize and solve the structure of full length M1 have, to date, been unsuccessful and consequently no high resolution model exists for the entire protein. The crystal structure of the 18 kDa NM domain (amino acids 1-164) has been determined at pH 4 [11]. Crystallization attempts at neutral pH on the full-length recombinant M1 protein produced in *E. coli* yielded crystals similar to those obtained in acidic media [8], [12]. However, in both acidic and neutral pH, only the structures of the NM-domains, that form dimers in the asymmetric unit, could be determined from the data.. The atomic structures determined at acidic and neutral pH display only minor differences with 1.2 Å root-mean-square deviation over the C_α_-positions [8]. Unfortunately, crystallographic studies have not yet provided structural information about the C-terminal domain of M1 nor information regarding pH structural changes.

A recent investigation of the full-length M1 in acidic solvent using bombardment with thermally-activated tritium atoms together with a number of experimental and bioinformatic methods [13] revealed that M1 is a structurally polarized molecule with a highly-ordered NM-fragment and a potentially unstructured C-terminal domain. These results suggest that the difficulties in the crystallization of the full-length protein may be related to the flexibility of the long C-terminal domain (residues 165 to 252).

Small angle X-ray or neutron scattering (SAXS or SANS), are methods used to analyse the low-resolution shapes of biological macromolecules in solution, and are applicable to the structural analysis of proteins with flexible regions [14]–[17]. In a SANS study of the full length M1 [12] the radius of gyration (*R_g_*) of 2.9 nm was found to be much larger than that expected for a 28 kDa globular protein. The protein length of 8.0 nm evaluated from the SANS data exceeded substantially the 6.0 nm size of the M1 rods observed under the viral membrane by electron microscopy (EM) [7]. To our knowledge, there are no other structural data on full length M1 in solution available, possibly due to difficulties of the protein preparation and its tendency to self-associate.

In the present work we analyze the overall structure of the full-length M1 and its NM-domain in solution at acidic pH by synchrotron SAXS. The protein conformation at acidic pH is perhaps most functionally interesting being related to one of the most puzzling properties of M1. The isolated M1 is soluble without detergents only at pH < 5.0 [18], the condition observed in the viral life cycle only at the very beginning of the infection when the interior of the virus is acidified prior to membrane fusion. In the cell at physiological pH, M1 exists solely in associate complexes with different viral and cellular partners [2], [4]. Knowledge about the structural properties of M1 allowing immediate decomposition of the layer inside the viral membrane during acidification is important for the understanding of the intermediate states of disassembly of the virion at early stages of infection. The structural information is expected to provide a key to a better understanding of the multi-functionality of the protein.

## Materials and Methods

### Influenza A virus preparation

Influenza virus strain A/Puerto Rico/8/34 (subtype H1N1) was propagated in 10-day-old embryonic chicken eggs and purified by centrifugation through 20% (vol/vol) sucrose in STE buffer (100 mM NaCl, 10 mM Tris-HCl, and 1 mM EDTA at pH 7.4) at 21,000 rpm for 90 min at 8°C in the SW 27.1 rotor of a Beckman-Spinco L5-75 centrifuge, as described in [19].

### Isolation of the M1 protein

The protein was isolated from intact influenza A/Puerto Rico/8/34 virions as described previously in detail [18]. The purity of the protein samples was determined by size exclusion chromatography, Coomassie and silver stained SDS-PAGE [20] and trypsin in-gel hydrolysis/MALDI-TOF mass spectrometry (see Figures S1 and S2). For further investigations, the M1 protein solution was dialyzed by Bio-Beards in 100 mM NaCl/20 mM MES buffer at pH 4.7.

### NM-domain isolation and purification

NM-domain was obtained by a trypsin proteolysis of the M1 protein solution (4 mg/ml) in NaCl/MES buffer, pH 4.7 for 30 min at 37°C using a protein/trypsin ratio 200∶1. The resultant solution was loaded onto a calibrated column Superdex G 75 Global (GE Healthcare), 10×300 mm, pre-equilibrated in 100 mM NaCl/20 mM MES buffer at pH 4.7. The column was eluted at 0.5 ml/min, and the eluted fractions were monitored at 280 nm. The single main elution peak corresponded to molecular mass of 18.1 kDa. The fraction was collected and its purity was analyzed by SDS-PAGE [20].

### Scattering experiments and data analysis

Synchrotron SAXS measurements were performed at the European Molecular Biology Laboratory (EMBL) on the storage ring DORIS III (DESY, Hamburg) on the X33 beamline [21] equipped with a robotic sample changer [22] and a PILATUS-1M detector (DECTRIS, Switzerland). The scattering intensity *I*(*s*) was recorded in the range of the momentum transfer 0.07<*s*<5.5 nm^−1^, where *s  =  *(4*πsinθ*)*/λ*, 2*θ* is the scattering angle, and *λ = *0.15 nm is the X-ray wavelength. The measurements were carried out in 100 mM NaCl/20 mM MES buffer, pH 4.7, with exposure times of 2 minutes in eight 15-seconds frames to monitor potential radiation damage (no radiation effects were detected). The data were corrected for the solvent scattering and processed using standard procedures [23]. To account for the interparticle interactions, solutions at M1 protein concentrations 4.3, 2.0 and 1.3 mg/ml, and at NM-domain concentrations 3.8, 3.0 and 1.5 mg/ml, were measured and the data were extrapolated to infinite dilution using PRIMUS [23].

The values of the forward scattering and radii of gyration *R_g_* were calculated from the experimental SAXS patterns using Guinier approximation,
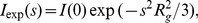
(1)


which is valid in the range of (*sR_g_*) approximately < 1.3 [14]. These parameters and the maximal diameter of the particle *D_max_* were also computed from the distance distribution function *p(r)*. The latter was evaluated by the program GNOM [24] using Eq (2)


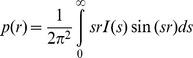
. (2)

The low-resolution shapes were reconstructed by two *ab initio* methods, DAMMIN [25] employing a dummy atom (bead) model of a particle, and GASBOR [26] employing a chain-like ensemble of dummy residues. Starting from a random assembly, both programs utilize simulated annealing (SA) to build models fitting the experimental data *I*_exp_(*s*) to minimize discrepancy
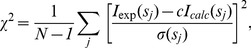
(3)


where *N* is the number of experimental points, *c* is a scaling factor and *I*_calc_(*s_j_*) and *σ*(*s_j_*) are the calculated intensity from the model and the experimental error at the momentum transfer *s_j_*, respectively.

In an alternative hybrid approach, approximate conformations of the missing C-terminal domain were reconstructed accounting for the high resolution structure of the NM domain (PDB entry 1AA7 [11]). The programs BUNCH [27] and CORAL [28] employ SA to search for the configuration of the C-terminal represented as a dummy residues chain to fit the computed scattering pattern from the full-length protein to the experimental scattering data. For both for *ab initio* and hybrid modeling, multiple reconstructions were performed, which yielded consistent models. The outputs were analyzed using programs SUPCOMB [29] and DAMAVER [30] to identify the most typical models best representing the spatial disposition of the NM-domain and full length M1 in solution.

The flexibility of the C-terminal was quantitatively assessed by the ensemble optimization method (EOM) [31]. This method selects an ensemble of possible conformations from a pool of randomly generated models, in this instance using, the crystal structure of the NM domain with a randomly generated C-terminal region. CRYSOL [32] was used to calculate the theoretical scattering from these models and a genetic algorithm (GAJOE) was employed to select ensembles of conformations whose combined scattering profiles best fit the experimental data.

To analyze the amount of aggregates in the M1 solutions, the program OLIGOMER [23] was used. Given the scattering intensities of components in a mixture, *I_i_*(*s*), the program fits the experimental scattering curve by their linear combination to determine their fractions *w_i_*. The equation 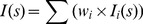
(4)


is solved with respect to *w_i_* by nonnegative least-squares to minimize the discrepancy between the experimental and calculated scattering curves.

The on-line size-exclusion chromatography (SEC-SAXS) experiment was performed at the EMBL beamline P12 on the storage ring Petra-3. A 45 µl aliquot of M1 protein was defrosted on ice and centrifuged at 30 000 × *g* for 20 min at 4°C. All subsequent manipulations were performed at room temperature. The sample (final concentration, 1.6 mg/ml) was injected onto a GE healthcare Tricon Superdex 75 10/300 24 ml column pre-equilibrated with 100 mM NaCl, 50 mM MES pH 4.0 at a flow rate of 0.4 ml/min. SEC-SAXS data measured in continuous flow mode from the SEC column eluates using 1-second exposure periods for a total of 3600 seconds (one column volume). Data were recorded using a Pilatus 2M pixel X-ray detector (Dectris), at a sample to detector distance of 3.1 m (0.05<*s*<4.0 nm^−1^) with a sample path length of 1.5 mm. The data was integrated and reduced to produce the radially averaged scattering profiles of each individual frame. Automated methods (Franke et al., in preparation) were used to initially assess the forward scattering *I*(0) of each frame and to calculate the *R_g_* as a function of frame number to identify those column eluates containing scattering particles. The automated analysis identified block of frames with forward scattering intensities and measurable *sR_g_* above those recorded for the background buffer (equating to an elution volume between 12–14 ml). The SEC-SAXS data frames spanning this region of the elution profile were scaled and subsequently averaged to produce the final scattering profile representative of M1 monomers in solution.

### Analytical ultracentrifugation (AUC) measurements and data analysis

Sedimentation velocity experiments were performed on a «Beckman E» analytical ultracentrifuge (USA) equipped with a scanner at a wavelength of 280 nm at rotor speeds of 60,000 rpm, 20°C. The program SEDFIT (sedfit12p52) was applied to model sedimentation profiles using the integrated Lamm equation solutions [33].

The relationships between the molecular mass *M_w_*, the sedimentation coefficient *s,* and the translational friction coefficient *f* were obtained using the Svedberg equation [34]:


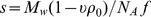
 (5).

The translational friction ratio *f*/*f_0_*, where *f_0_* is the translational friction coefficient for a sphere of the same mass, was determined by [34]:


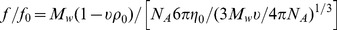
 (6).

Solvent density *ρ_0_*, viscosity *η_0_* and the specific partial volume *υ* of the M1 protein were calculated at 20°C by SEDNTERP (http://www.jphilo.mailway.com/). Intrinsic sedimentation coefficients (*s_20,w_*) corrected to water at 20°C were calculated by SEDNTERP from the experimental *s* values obtained in the appropriate solvent.

### Atomic force microscopy (AFM)

Structures formed by M1 protein upon adsorption on a negatively charge mica surface was studied on the Multimode Nanoscope IV setup (Veeco Digital Instruments, USA) equipped with E type scanner and electrochemical fluid cell. All experiments were performed in tapping mode at room temperature in a buffer solution. Sharpened SiN_3_ cantilevers with nominal spring constant of 0.06 N/m (Veeco Digital Instruments, USA) and tip radius of approximately 1 nm were used for scanning. A 200 µl droplet of 10 µg/ml protein solution was placed onto the surface of the disk of freshly cleaved mica with diameter of 1.5 cm (Veeco Digital Instruments, USA). The sample was incubated for 1 hour at room temperature, rinsed 5 times with working buffer solution (100 mM NaCl/20 mM MES, pH 4.7) and put into the AFM cell filled with the same working buffer for scanning. AFM images were processed using WSxM software [35].

## Results

### Overall characteristics of M1 protein and its NM-domain

**Figure 1:**
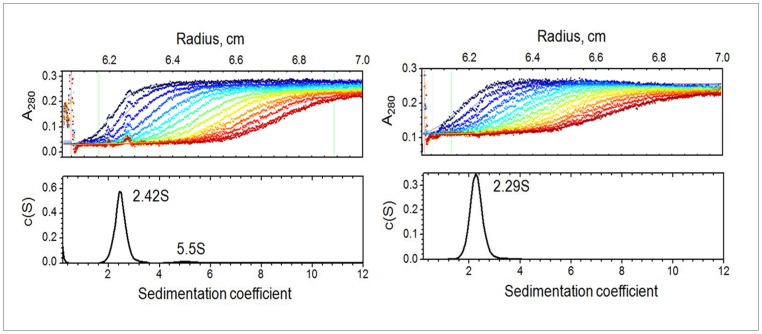
Sedimentation velocity experiment on the M1 protein (left) and the NM-domain (right). Top panels, raw data; absorbance at 280 nm plotted as a function of the radial position, every fifth curve is shown; lower panels, fitted distributions of the sedimentation coefficients.

The overall characteristics of the full-length M1 protein and its NM-domain in solution were first assessed with AUC. Both specimens yield sedimentation profiles with well defined monomeric peaks, but for M1 a minor peak was also observed at 5.5 *S* corresponding to large associates (Figure 1). These associates are well separated in size from the monomers and comprise a minor fraction of about 2–3 volume percent. The sedimentation velocity experiments also provide evidence for the structural anisotropy of M1. Although the NM-domain *f*/*f_0_* was 1.06 indicating a nearly spherical shape for this region of the protein, the translational friction ratio *f*/*f_0_* of 1.27 obtained for the full-length M1 indicates a noticeable deviation from a spherical shape. Furthermore, the distribution of the sedimentation coefficients for the full-length M1 centered at *s_20,w_* of 2.42 *S* is significantly lower than the theoretical value for a globular protein consisting of 252 amino acids (*s_max_* = 3.15 *S* ). These results suggest that M1 protein adopts a rather anisometric shape of in solution.

**Figure 2:**
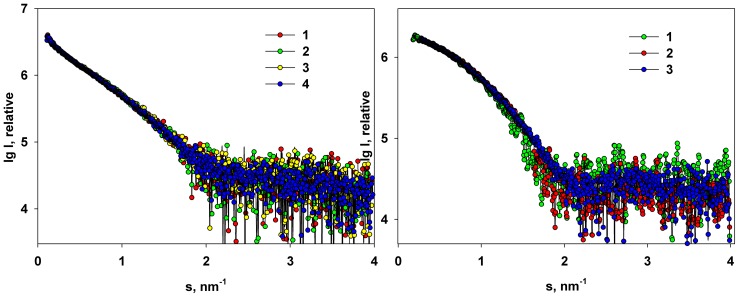
Experimental SAXS patterns from the full length M1 protein (left panel) and the NM-domain (right panel). The individual curves correspond to the varying solute concentrations; for M1, curves 1 to 4 represent c =  4.5 mg/ml; 3.4 mg/ml, 2.3 mg/ml and 1.7 mg/ml, respectively; for NM-domain, curves 1 to 3 represent c = 3.8 mg/ml, 3.0 mg/ml and 1.5 mg/ml, respectively.

Further structural characterization of M1 and NM was performed by SAXS. The experimental data from M1 at all concentrations reveal an upturn at very small angles (Figure 2, left panel), while those from the NM-domain do not display this behavior (Figure 2, right panel). Both constructs showed no concentration dependence of the scattering data and the appropriately scaled curves were superimposable for all measured concentrations. These results indicate that monomeric M1 particles co-exist in solution with associates that are formed independently of the solute concentration and reflect the propensity of M1 to associate even at acidic pH. The NM-fragment on its own does not form aggregates or clusters under these conditions.

**Figure 3:**
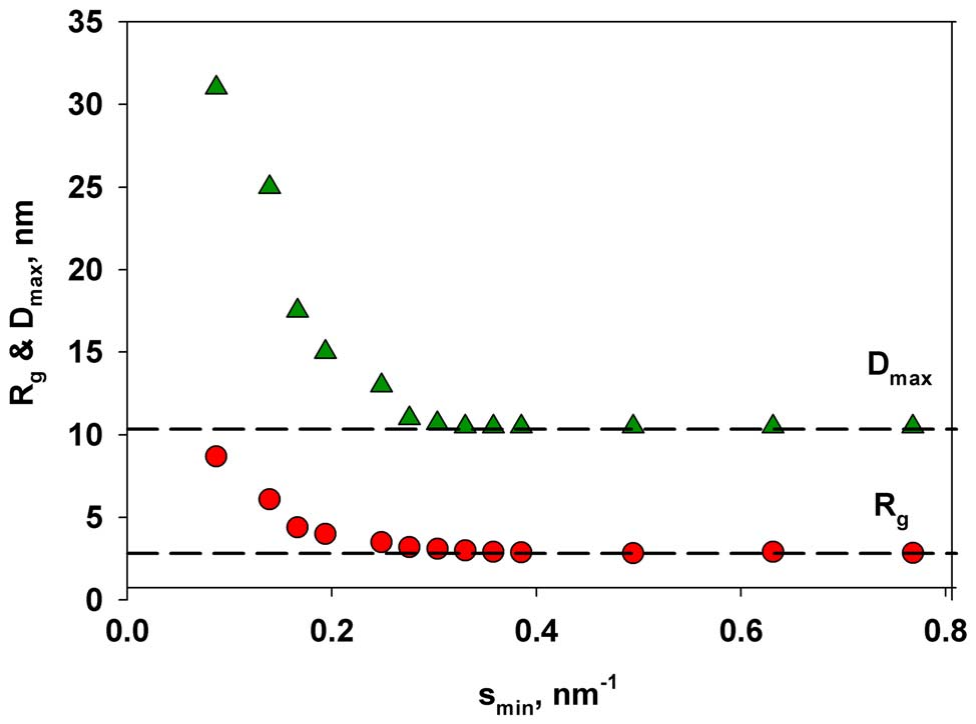
The *R_g_* and *D_max_* computed from the solution of the full length M1 protein as a function of the cut-off value at small angles *s_min_*.

The absence of the concentration dependence and clear separation between the monomeric fraction and the aggregates open the possibility to minimize the contribution of the latter species to the scattering and to construct the structural model of the monomeric full-length M1. This can be done by calculating the distance distribution function *p*(*r*) of the solute using the indirect transformation [24]. As the large aggregates contribute mostly to the very small angles, discarding this portion of the data diminishes the influence of these particles. A systematic removal of the increasing portions of the low angle data in the GNOM [24] analysis indicated that starting from the cut-off value of *s_min_*≈0.3 nm^−1^ the *R_g_* and *D_max_* reach a plateau (Figure 3). This result suggests that in the range of the scattering data beyond *s_min_*≈0.3 nm^−1^ the influence of the aggregates is negligible and this range can be employed to analyze the structural properties of the M1 monomers.

**Figure 4:**
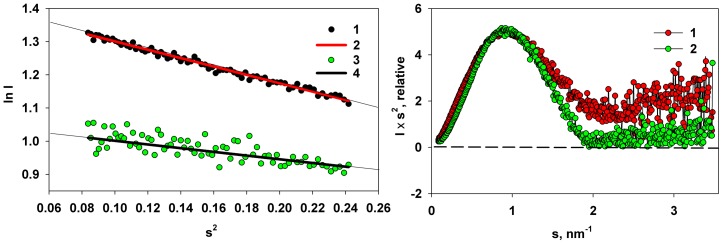
Guinier and Kratky plots for the M1 protein and for the NM-domain. Left panel: Guinier plots of M1 (1 – experimental data; 2 – Guinier fit) and of the NM-domain: (3 – experimental data; 4 – Guinier fit). Right panel: Kratky plots for M1 (1) and NM-domain (2).

The scattering data from M1 without the very low-angle aggregate data show a linear correlation in the Guinier region for the scattering vector range 0.3 < *s* < 0.44 nm^−1^ (Figure 4, left panel) yielding *R_g_* = 2.9±0.1 nm. This range corresponded to *sR_g_* limits from 0.87 to 1.29 and the computed *R_g_* agrees well with the one previously reported [12]. The MM of the solute obtained from the extrapolated *I*(*0*) value, 27±3 kDa, corresponds well to the calculated MM of full-length M1 based on the amino acid sequence (27.9 kDa). These overall parameters confirm that discarding the initial portion of the scattering data allows one to remove the contribution of the large aggregates to the scattering from the solution of M1.

The *R_g_* of the NM-domain (1.9±0.1 nm) and the MM of the NM construct (17±2 kDa) derived from the Guinier approximation in the same angular region as for M1 is much smaller than that of the full length protein (Figure 4, left panel). These data indicate that the NM-domain is monomeric in the acidic solution. The MMs of both the full-length M1 and its NM-fragment estimated by sedimentation velocity in Figure 1 (27.9±1.4 kDa and 18.5±1.0 kDa, respectively) fully agree with the SAXS data.

The Kratky plots of the two constructs (Figure 4, right panel) have a characteristic bell-shaped appearance indicating that both samples are mostly folded [14]–[17], [36]. However, the plot for M1 displays elevated scattering at higher angles suggesting that part of the protein (presumably C-terminal) may be flexible. In contrast, the Kratky plot of the NM domain appears typical for a compact globular protein. The excluded (Porod) volumes of M1 and of the NM domain are 55±7 nm^3^ and 36±5 nm^3^, respectively. Given that the empirical ratio between Porod volume and molecular mass of a protein is about 1/(1.7÷2.0) [36], these volumes are compatible with the expected MMs of the monomeric constructs for both cases.

### *Ab initio* shape restoration and the analysis of flexibility of the M1 C-terminus

**Figure 5:**
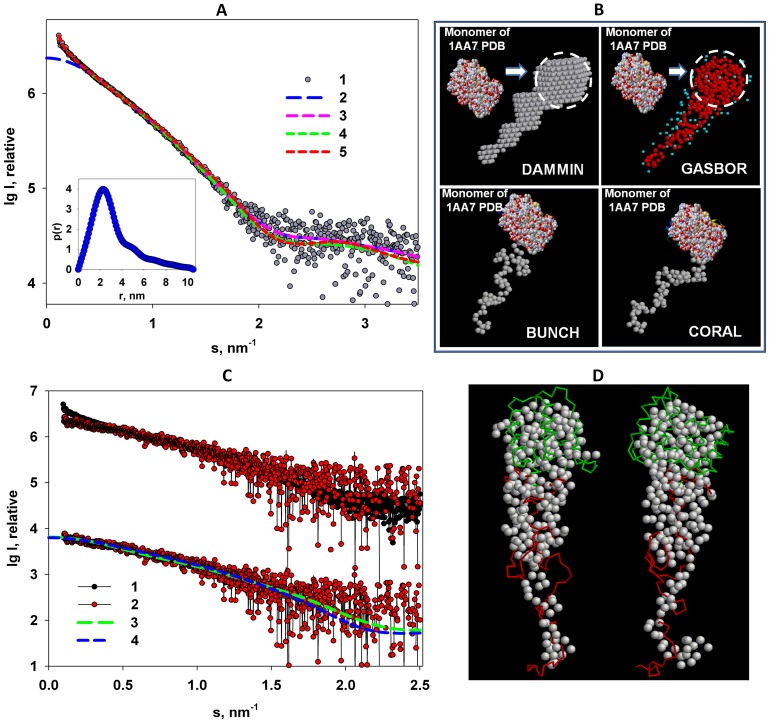
Shape restoration of full length M1. (A) Experimental SAXS data (1), the transformed from *p*(*r*) and extrapolated to zero scattering angle intensity (2), scattering patterns computed from the *ab initio* models (DAMMIN & GASBOR) in (B), top row (3), scattering patterns computed from the hybrid models (BUNCH & CORAL) in (B), bottom row (4), and the fit from the mixture computed by OLIGOMER (5). Insert: distance distribution function *p*(*r*) computed by GNOM. (B): structural models reconstructed by *ab initio* and by hybrid modeling programs. (C): Comparison of the experimental SAXS curve from M1 protein (1) with the SAXS profile that after passage of M1 through the chromatographic column (2), and the scattering patterns computed from the hybrid models in panel B: CORAL (3) and BUNCH (4). (D) Superposition of the *ab initio* model restored by GASBOR from the SAXS-SEC data (beads) with the BUNCH model reconstructed from the truncated raw M1 protein data (C_α_ chain; green: N domain, red: C-terminus). The models represented on the left are a 90° rotation of the models on the right.

The distance distribution function *p*(*r*) of M1 computed by GNOM using the intensity truncated at *s* = 0.3 nm (Figure 5, left panel, insert) yields the maximum size *D_max_* of 10.5±1 nm. The skewed profile of the *p*(*r*) suggests that the protein has a globular region with a size of about 4 nm (presumably, the NM domain) and an extended tail with the length of about 6 nm (presumably, the C-terminal region).

**Figure 6:**
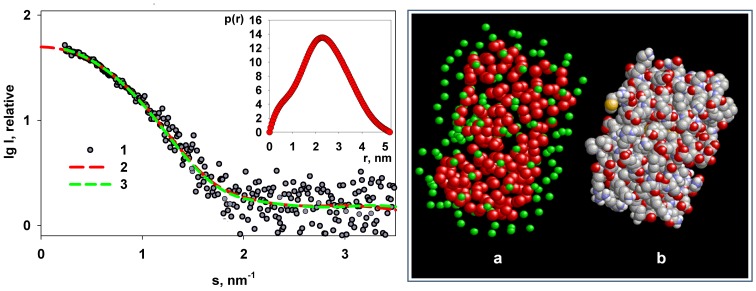
Shape restoration of the NM-domain. Left panel: experimental SAXS data (1), the transformed from *p(r)* and extrapolated to zero scattering angle intensity (2), scattering pattern computed from the GASBOR model (3). Insert: distance distribution function *p(r)* computed by GNOM. Right panel: the model reconstructed by GASBOR (red balls, dummy residues, green balls: dummy water molecules) (a), crystal structure of the NM-domain (PDB code 1AA7) (b).

The low-resolution shape of M1 reconstructed *ab initio* using DAMMIN [25] and GASBOR [26] both yield good fits to the experimental data (Figure 5A) with discrepancy *χ* ≈ 1.2. Typical models displayed in Figure 5B (top row) contain a compact globular region and an extended tail. The shape of the globular part indicated by circles in Figure 5B agrees well with that of the crystal structure of the monomeric NM domain (PDB code 1AA7). The *ab initio* low-resolution shape of the NM fragment neatly fits the experimental data (*χ* ≈ 1.2) and the shape is fully compatible with the crystallographic structure of the monomeric NM domain (Figure 6).

**Figure 7:**
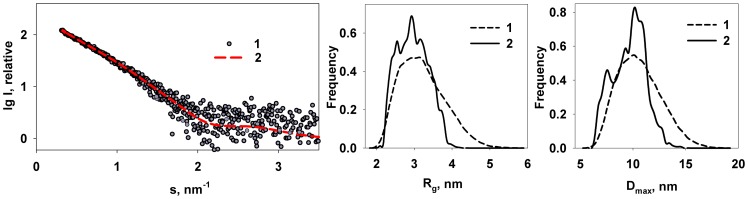
Flexibility of the C-terminal of M1 analysed by EOM. Left panel: experimental SAXS data (1) and the scattering from the selected ensemble. Middle and right panels: *R_g_* and *D_max_* distributions, respectively (random pool (1), selected ensemble (2)).

The *ab initio* modelling strongly suggests that M1 contains a globular NM-fragment similar to that of the NM crystal structure connected to an extended, perhaps weakly ordered C-terminal domain. The flexibility of the latter domain was quantitatively assessed by EOM [31] as described in Methods. The final sub-ensembles of full length M1 that were selected from initial pool of random structures provide a good fit to the experimental data with *χ* ≈ 1.2 (Figure 7, left panel). The *R_g_* and *D_max_* distributions of the selected EOM ensembles are substantially narrower than those of the initial pool (Figure 7, middle and right panels) and hyper-extended structures with *R_g_* and *D_max_* exceeding 4 nm and 13 nm, respectively, are absent in the final ensembles. These results indicate that the C-terminal, although extended, does not behave like a completely random chain but instead has a limited flexibility. The maximum particle size in the selected M1 ensembles varies from 6 nm to 13 nm with the most abundant size of about 10–11 nm. Given that the diameter of the NM domain is 4 nm, the extent of the C-terminal domain spans the range between 2 nm and 9 nm with the most populated length of about 6–7 nm.

The limited flexibility revealed by EOM allowed us to further try and visualize the average conformation of the C-terminus employing hybrid *ab initio* and rigid body modelling (programs BUNCH [27] and CORAL [28]), that model the C-terminus in the form of a dummy residue linked to the crystal structure of the NM domain. The two programs yielded consistent models that fit the experimental SAXS data from the full length M1 with *χ* ≈ 1.3 (Figure 5A). These hybrid models also show that the C-terminus extends from the NM domain and are, overall, compatible with the shapes determined by *ab initio* reconstructions (Figure 5B, bottom row).

To further validate the model of M1, we have also performed an on-line SEC-SAXS experiment allowing one to separate components of different size in a mixture. In this experiment, a SEC column was directly connected to the SAXS beam line and about 3000 scattering patterns were recorded from the elution profile (see Methods). The set of the scattering patterns corresponding to the monomeric M1 peak were detectable in the SEC-SAXS data. Although the signal averaged over the monomeric fraction is rather noisy because of an approximate ten-fold dilution that occurs during the experiment, the scattering pattern agrees with SAXS data previously collected from M1 without SEC except for the very small angles (Figure 5C). The data after the on-line purification yield *R_g_* = 2.7±0.2 nm, which is in good agreement with the value deduced from the non-SEC data after removing the small angle portion. The shape of the monomer reconstructed *ab initio* from the SEC-SAXS data also agrees well with the hybrid models constructed from the truncated scattering from M1 (see overlap in Figure 5D). Moreover, the scattering computed from the CORAL and BUNCH models in Figure 5B fits well the experimental SEC data (Figure 5C). Therefore, the analysis of the on-line purified monodisperse solution of M1 further corroborates the structural models obtained from the original M1 solutions.

The SAXS results indicate that the NM-domain of M1 adopts a compact globular state in solution while the C-terminus is, first, extended, and second, exhibits a limited flexibility allowing different conformers of the protein co-exist in solution. These C-domain chain conformations display variable lengths, and one may speculate that the length of the C-terminus is a factor regulating the interaction modes of M1 under different conditions.

### Shape analysis of the M1 self-assemblies

**Figure 8:**
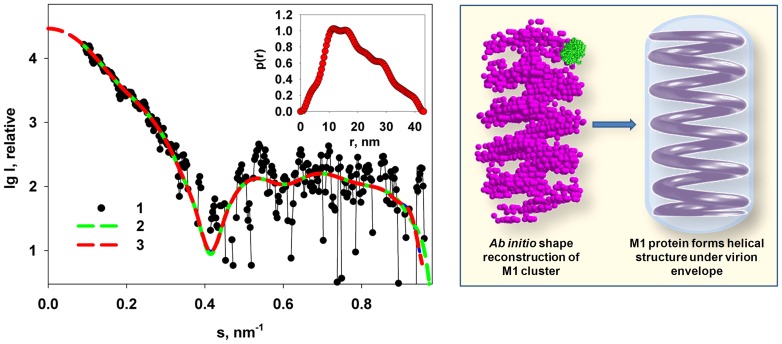
Shape restoration of the M1 clusters. Left panel: difference curve corresponding to the scattering from the clusters (1), the transformed from *p*(*r*) and extrapolated to zero scattering angle intensity (2), and the scattering from the *ab initio* DAMMIN model (3) Insert: the distance distribution function of the clusters. Right panel: typical reconstructed model of the cluster by DAMMIN and hypothetical model of the self association of the M1 protein to maintain the virion envelope. The crystallographic model of the NM domain protein is shown in green for size comparison.

The observed upturn in the low angle scattering data from M1 solutions (Figure 2) was concentration-independent suggesting that the structural aggregates (clusters) of M1 possess a defined structure. It is conceivable that such clusters may serve as nuclei in the M1 self assembly process and we have therefore attempted to get further insight into their organization. To obtain the scattering from the clusters, the computed scattering from the *ab initio* model of the individual M1 particles was subtracted from the experimental data of the full-length protein. The difference curve (Figure 8, left panel, curve 1) was used for the analysis of the clusters.

Using GNOM, the *R_g_* was found to be 13.5±0.5 nm, and the distance distribution function *p*(*r*) of the clusters presented in the insert of Figure 8 has a *D_max_* = 43±5 nm. The *p*(*r*) function displays several shoulders, which suggest a regular internal structure of the clusters. The *ab initio* shape of the clusters was reconstructed using DAMMIN, whereby multiple runs yielded reproducible results indicating a stable shape restoration. A typical *ab initio* shape presented in Figure 8 (right panel) shows that the M1 clusters have a quasi-helical organization with the average layer thickness of about 6 nm coinciding with the size of M1 in densely packed layers [7]. The overall conformation of the clusters is similar to that predicted by Harris et al [8] and observed by Calder et al in electron cryo-tomography experiments in authentic influenza virions under the viral envelope [9]. One can conclude that the intrinsic property of M1 to self-assemble in solution, even at acidic pH, is biologically determined, being related to its function as a matrix protein. We believe that the obtained helical structure may be a prototype of a native matrix layer formed by M1 protein under the virion envelope. Its flexibility, similar to any helical (coil) flexibility, allows for changes of the shape and size of the virion during the virus life cycle and in the process of cell infection.

Given the two *ab initio* models of the individual M1 monomers and of the clusters, the experimental SAXS data from M1 containing both species were fitted by a linear combination of their scattering patterns. The best fit as calculated with OLIGOMER [23] yields the volume fraction of clusters of 0.03±0.01, which agrees well with the AUC experiments. The mixture yielded a good fit to the experimental data in the entire measured range (*χ* = 1.04, Figure 5, curve 5).

**Figure 9:**
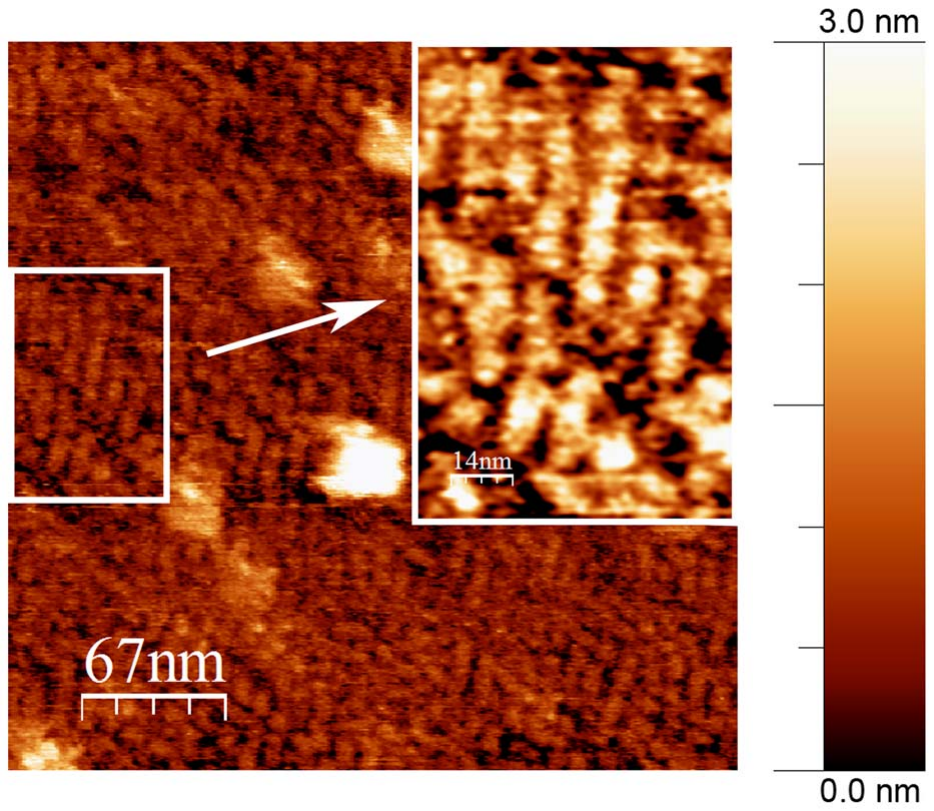
AFM topography image of M1 protein structures formed at negatively charged surface. The inset displays a magnification of the region outlined by white frame.

Additional evidence for M1 self-assembly at low pH was obtained by AFM. As the adsorption of M1 on a lipid bilayer is predominantly electrostatic and requires a negatively charged net of the lipid membrane [7], the negatively charged mica [37] serves as a good model of a lipid bilayer. The AFM topography images (Figure 9) reveal that the protein forms ordered stripes having an average thickness and length of about 6.4±0.7 nm and 35±5 nm, respectively. These results correlate well with the SAXS models in as much as the particles observed in AFM could serve as precursors in the formation of the M1 quasi-helices. Similar M1 assemblies with layered arrangements could also be observed on negatively stained protein preparations by transmission EM (Figure S3). All our results therefore reveal a biologically determined tendency of M1 to self-assemble into helix-like structures both in solution and on a lipid-like negatively charged surface.

## Discussion

In the present study, SAXS, AUC and AFM are employed to characterize the overall structure and association behaviour of full length M1 at acidic pH. This condition occurs in the very beginning of cell infection leading to an acid-triggered fusion of the viral membrane. Low pH has been previously found to serve as a switch that allows M1 to carry out its multiple functions in the uncoating, nuclear transport, and assembly of the viral ribonucleocapsid [38]. Structural insights into M1 at low pH are therefore of major importance in understanding the functional properties of the protein during one of the most crucial stages of the virus life cycle.

For most SAXS studies the effects of aggregation must be avoided as they hamper the interpretation of the experimental data in terms of 3D models. In this particular case, the M1 clusters are much larger in size than the monomers, which makes is possible to meaningfully separate the contribution of the two species. The validity of this procedure is further corroborated by the on-line SEC-SAXS experiment allowing us to observe the scattering intensities from a monodisperse solution of monomeric M1, albeit at rather low concentration. Overall, our findings point to the structural anisotropy of the M1 macromolecule, whose shape contains a globular core (NM-domain) and a moderately flexible tail (C-terminus). These SAXS results are supported by our sedimentation velocity experiments that provide further evidence that M1 adopts an anisotropic shape in solution.

The proposed structural model of the full-length M1 in acidic solution is also in keeping with the results derived from tritium planigraphy [13]. In this method, the radioactive label distribution along the polypeptide chain characterizes the steric accessibility of the target for the bombarding tritium atoms, and the most exposed parts of the macromolecule are the most intensely labeled. The highest level of tritium label incorporation was detected for the C-terminal domain (45.2%) and in the inter-domain loops (21.8 and 14.4%), while the N and M domains showed low labeling level (10% and 8.6%, respectively). In addition to suggesting structural anisotropy of M1 these results indicated that the translocation of M1 from a membrane-bound shell to the acidic solution in late endosomes may cause a pronounced conformational change, especially in the C-terminal domain and in the inter-domain region between the N- and M-domains.

The observed flexibility of the C-terminus – aside from being a probable explanation for unsuccessful crystallization attempts – may be considered an essential feature responsible for the multi-functionality of M1, in particular, for its ability to mediate the multistep process of cell infection. In contrast to full length M1, the isolated NM-domain is compact and does not reveal tendency to self associate, further emphasizing the key role of the C-domain in the interactions within the virion and in the formation of the well ordered helical macro-structure [9]. Indeed, the ability of M1 to self-associate that has been previously analyzed using M1-M1 stacking and M1-M1 twofold interfaces [8] yields a helical super-structure with an outer diameter of approximately 20 nm that is remarkably similar to the cross section of our SAXS-based *ab initio* reconstruction of the M1 clusters shown in Figure 8. Our results also correlate well with the previous EM observations of the free M1 oligomers and of the M1 layer within the virion [39]–[41]. Moreover the present results may help to gain a better understanding the process of M1 self-assembly. There is an evidence that M1 alone can form virus-like particles (VLPs) [42], [43], whereas other studies indicate that M1 cannot form VLPs on its own and other viral proteins are required, in particular, M2 [44], [45]. Our data suggest that self-assembling is an intrinsic biological property of the M1 protein, both in solution and on the lipid bilayer.

The AFM experiments reveal well ordered stripe-like structures formed by M1 on a membrane mimetic surface that correlate with previous observations [7], [8]. These formations could be treated as pre-helix protein superstructures, whose assembly could be triggered by the negative surface charge. Generally, the cluster visualized by SAXS and AFM may thus represent a template of higher order M1 associations formed by the interactions with other components within the virion. The formation of clusters is also corroborated by negative staining EM experiments (Figure S3). We should point out that the results of AUC, AFM and EM should be considered in the context of potentially dynamic objects like the M1 supramolecular assemblies. Although all of these techniques involve some (more or less specific) treatment of the sample that may or may not perturb the organisation of M1 during sample handling, our SAXS investigation performed on ‘simple’ dilute solutions of M1 and its assemblies go toward reinforcing the results from AUC, AFM and EM and provide the requisite synthesis between these biophysical techniques in regard to the structural analysis of M1 protein and its self-assembly.

Partial disorder and flexibility of the C-domain with many exposed regions [13] makes the entire M1 an amphitropic molecule, which displays affinity for both aqueous and hydrophobic environments promoting multiple protein-lipid and protein-protein interactions in different interaction modes [4]–[6]. The self-association of M1 protein that we observe in acidic solutions may reduce the configurational entropy, so that a cooperative structural transition upon self-assembly possibly takes place in both NM- and C- domains following a synergistic mechanism. We hypothesize that such cooperative transition is an essential characteristic of M1 allowing the protein to compose and maintain the viral matrix shell, and to be responsible for its multi-functionality and for viral intrinsic structural variability (pleiomorphy). The structural results for M1 reported here are commensurate with the overall biophysical features of many viral proteins. Viral proteins – and RNA virus proteins in particular – possess a high proportion of disordered fragments and loosely packed cores [46]. These features may promote ability of the viral proteins to interact with the host components as well as increase the adaptability levels and mutation rates of the viruses.

In conclusion, the results of the present work reveal the structural peculiarities of the full-length M1 protein as key adaptor molecule of the influenza virus. Our findings provide the basis for a further analysis of the hierarchy of M1 interactions and the development toward an ultimate deactivation of influenza A viral particles.

## Supporting Information

Figure S1 Size exclusion chromatography (SEC). Purified M1 protein elution profile on a Superdex G 75 Global column.
(TIF)

Figure S2 Gel electrophoresis. Coomassie (left) and silver (right) stained SDS-PAGE. Fractions during PAGE analysis were separated by discontinuous Tris-Glycine 15% SDS-PAGE. Aliquots of 25mkg (1) and 1 mkg (2) of purified M1 protein, and 5 mkg (3) of purified influenza virus per lane were analyzed. Both HA and NP fractions are absent on the purified M1 protein lanes. Additionally, M1 protein identity was confirmed by trypsin in-gel hydrolysis/MALDI-TOF mass spectrometry analysis (sequence coverage was equal to 55 to 71%).
(TIF)

Figure S3 Electron microscopy (EM) experiment. A typical EM image of M1 clusters. M1 protein forms on the grid surface agglomerates of virus-like particles with sizes of about 20 - 40 nm (left panel). A magnified image of a virus-like particle displaying a layered structure is shown on the right panel. The sample for the transmission EM was prepared by spotting the dialyzed M1 protein solution in MES/NaCl buffer, pH 4.0, at the concentration of ∼ 0.02 mg/ml onto Formvar-coated grids. Contrast was adjusted by adding 2% aqueous solution of phosphotungstic acid (Fluka) at pH 7.5. The grids were then analyzed using a Jeol JEM-1400 electron microscope.
(TIF)
