# Supplementary figures and images for: Structural Analysis of Influenza A Virus Matrix Protein M1 and Its Self-Assemblies at Low pH
Source: PLoS One. 2013 Dec 16;8(12):e82431. doi: 10.1371/journal.pone.0082431 (PMC3865061; doi:10.1371/journal.pone.0082431)

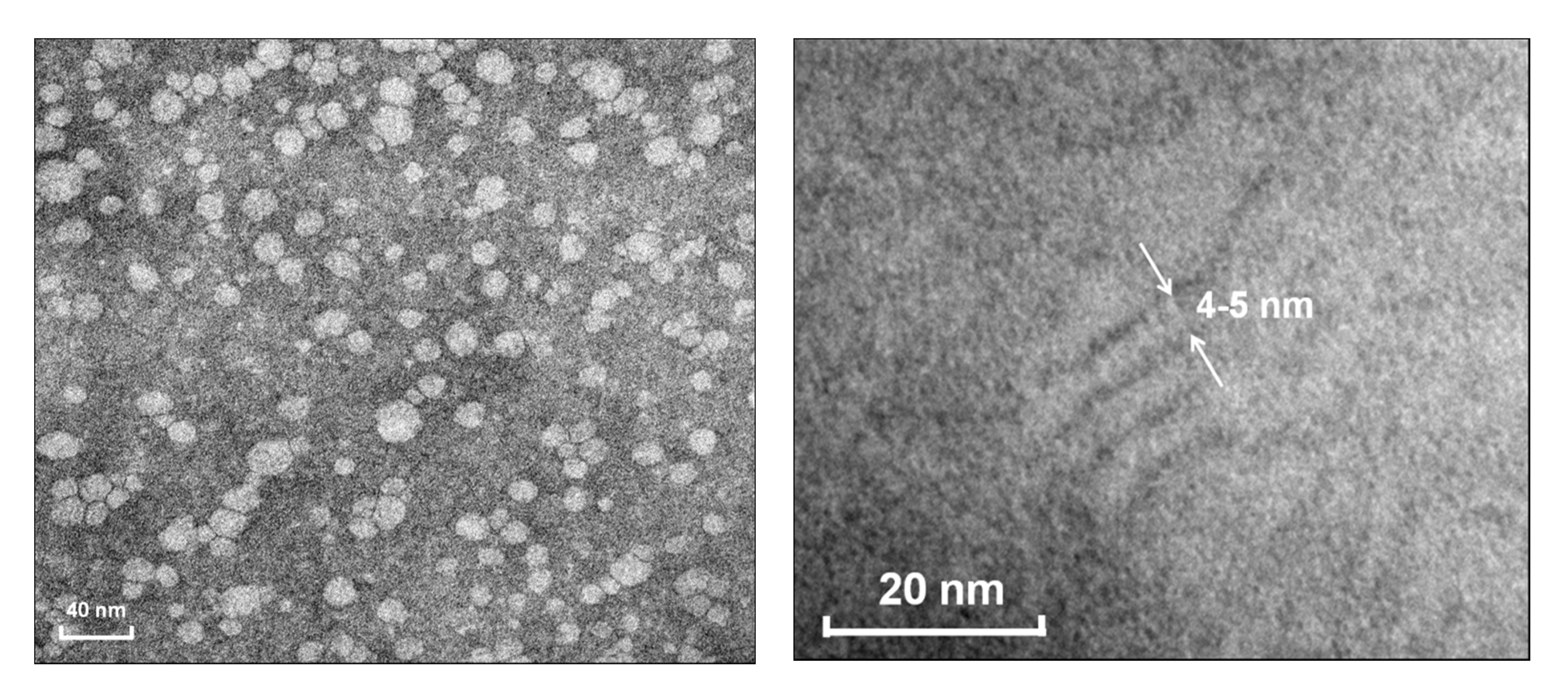

Supplement: Figure S3 — Electron microscopy (EM) experiment. A typical EM image of M1 clusters. M1 protein forms on the grid surface agglomerates of virus-like particles with sizes of about 20 - 40 nm (left panel). A magnified image of a virus-like particle displaying a layered structure is shown on the right panel. The sample for the transmission EM was prepared by spotting the dialyzed M1 protein solution in MES/NaCl buffer, pH 4.0, at the concentration of ∼ 0.02 mg/ml onto Formvar-coated grids. Contrast was adjusted by adding 2% aqueous solution of phosphotungstic acid (Fluka) at pH 7.5. The grids were then analyzed using a Jeol JEM-1400 electron microscope. (TIF) [file pone.0082431.s003.tif]
